# Anxiety, Depression and Quality of Life in Patients with Neuroendocrine Neoplasia After Surgery

**DOI:** 10.1007/s00268-022-06479-z

**Published:** 2022-02-22

**Authors:** Nehara Begum, Hannah Hunold, Berthold Gerdes, Tobias Keck, Annika Waldmann

**Affiliations:** 1 Department of General-, Visceral-, Thoracic- and Endocrine Surgery, Johannes Wesling Klinikum Minden, University Hospital of the Ruhr-University Bochum(RUB), Hans-Nolte Str. 1, 32429 Minden, Germany; 2 Institute of Social Medicine and Epidemiology, University of Luebeck, Luebeck, Germany; 3 Department for General Surgery, University Hospital Schleswig-Holstein (UKSH), Ratzeburger Allee 160, 23538 Luebeck, Germany

## Abstract

**Background:**

Neuroendocrine neoplasia (NEN) are rare and complex, with surgery as key therapy even in cases with metastasis. Little is known regarding the quality of life, prevalence of depression, anxiety and the impact of surgery.

**Methods:**

This prospective, follow-up study included 90 consecutively recruited patients with NEN after surgery in a university hospital. The EORTC QLQ-C30, EORTC QLQ-GI-NET.21, and Hospital Anxiety and Depression Scale and a hospital specific questionnaire were completed during follow-up after 3 to 5 years (t1–t5).

**Results:**

Mean age was 54 (SD 15) years, 13% had secondary malignancies and 11% had psychiatric diagnoses (depression *n* = 8, schizophrenia *n* = 2) pre-existent. Critical life events occurred in 51% within 5 years before diagnosis. Surgery was done in curative intention in 82% and R0-resection rate was 90%. The median survival was 25.3 years. The 10-year survival rate was 87%, 98%, 95% and 26% for all patients (*n* = 90), stage I/II (*n* = 45), stage III (*n* = 25) and stage IV (*n* = 20), respectively (*p* < .001). Anxiety score was pathological in 30% after 1 year (t1) and in 10% after 5 years, depression score in 25% (t1) and 30% (t5). Fatigue and muscle/body pain were elevated symptoms with > 50 and 40 points 3 years after surgery.

**Conclusion:**

Depression rate remains high whereas anxiety declines over time. Fatigue and muscle/body pain were identified as relevantly elevated after surgery. Systematic screening and supportive therapy should be implemented during follow-up after surgery.

## Introduction

Neuroendocrine neoplasia (NEN) of the gastrointestinal tract are a rare and heterogeneous tumour entity with estimated incidence of 4–6/100,000 per year. Survival rates are high even in metastatic disease resulting in a prevalence of 35/100,000 per year [1]. Median age at diagnosis is in the sixth decade although many patients with inherited syndromes are younger. Around 50% of the patients have distant metastases at time of diagnosis and up to 80% show locoregional lymph node metastases [2, 3]. Surgery is the key therapeutic option even in the presence of distant metastases and so far the only curative treatment. According to data of the German NET-Registry, surgery is applied up to the sixth line therapy along a NEN-patient career [2, 4]. Prognostic factors are tumour stage at diagnosis, primary localisation and tumour grading, dependent on Ki-67%, according to ENETS-proposal [5–7]. Since 2009 the TNM-classification for NEN was adopted to the ENETS-proposal and leaded to a practicable and widely accepted classification system [8–10].

Many NEN are slow-growing, chemotherapy-resistant G1- or G2-NET with a proliferation-index of Ki-67 < 20%. Even after complete and curative tumour resection a risk of recurrence remains. Tumour recurrences can occur even 10 to 20 years after curative resection [11, 12].

Functional activity is seen in around 20% of NEN-patients, most often carcinoid syndrome due to systemic hypersecretion of serotonin in case of hepatic metastases of GI and pulmonal NEN [13]. Psychiatric dysbalances like depressivity and sleeping disorders are described in 3–10% of the patients with carcinoid syndrome [14]. The serotonin-synthesis of functional active NEN may result in a tryptophan deficiency. Tryptophan is an essential amino-acid, which is generally converted into niacin and the precursor of multiple bioactive products. It is estimated that up to 60% of the resorpted dietary tryptophan may be consumed in case of small bowel NEN by the neoplasia itself [15, 16]. Deficiency of essential amino acids might influence the psychological status of the patients as well [17, 18]. The role of the central serotonergic system is not fully understood, yet the dysfunction of this system is associated with depression, anxiety, sleeping disorders, impaired social and cognitive function [19, 20]. Depression and anxiety are a major issue in cancer patients and can affect the immune system [21, 22]. This results in an impaired immune response by modulating the secretion of pro-inflammatory cytokines and thereby the response to infections and tumour response [23–25].

So, several mechanisms which influence quality of life, depression and anxiety are possible in NEN: these are long-term effects of surgery, lifelong tumour burden, danger of recurrence and possibly the lack and deficiency of essential amino acids in functional active NEN [16]. While data for quality of life (QoL) exist for advanced NEN from clinical studies, no data exists for NEN treated with surgery in curative intention [26, 27]. Aim of this study was to elucidate 1. The prevalence of pre-existent psychiatric comorbidities and 2. To describe anxiety, depression and quality of life in a surgically treated cohort of NEN-patients.

## Materials and methods

Patients for this study were consecutively recruited after surgery for NEN in the department for surgery at the university hospital Schleswig–Holstein (UKSH), Campus Luebeck, at the first follow-up visit after surgery in the outpatient clinic. Recruitment period was from 01/2009 to 12/2015. All patients had to give written consent for participation in the local NEN registry. Recruitment started within 3 month after surgery to reduce bias driven by anxiety and excitement before surgery and study questionnaire was completed at each further follow-up visit. The study questionnaire included questions on clinical aspects, sociodemographic data and validated questionnaires for assessing anxiety and depression (HADS-D) and health-related quality of life (EORTC QLQ-C30) and since 2012 EORTC QLQ-Gi.NET 21. The third battery of hospital specific questionnaires included questions on important life events (such as death of a beloved, divorce, serious diseases), on additional cancer treatment (radio-, / chemo-, immunotherapy), use of complementary alternative medicine and on psychological support (psychotherapy, patient groups). Time periods were defined as t1 = 3–12 month, t2 = 13–24 month, t3 = 25–36 month, t4 > 36 and t5 > 60 month after surgery. For HADS-D t1 to t5 are available, for EORTC QLQ-C30 and GI.NET 21 t1–t3 is available only, because this questionnaires were introduced later.

### Hads-D

The German version of the Hospital Anxiety and Depression Scale (HADS-D) consists of 14 items in total, of which each seven are grouped to the subscales anxiety and depression. The four possible answers per item are coded zero (absence of problem) to three (most severe problem); thus, the subscale scores range from zero to 21. The higher the score, the higher is the level of anxiety and depression. A score of eight to ten indicates at least borderline anxiety, a score over 10 a manifest anxiety and depression [28].

### EORTC QLQ-C30 and EORTC QLQ-GINET21

The QLQ-C30 consists of 30 items covering five function subscales (physical, role, emotional, cognitive, social function C30), nine symptom subscales/items (fatigue, nausea/vomiting, pain, dyspnoea, insomnia, appetite loss, constipation, diarrhoea, financial difficulties) and a global health/QoL subscale [29]. The GINET21 includes 21 items covering four single-item assessments relating to muscle and/or bone pain (MBP), body image (BI), information (INF) and sexual functioning (SX) as well as 17 items organised into five symptom scales: endocrine symptoms (ED; 3 items), GI symptoms (GI; 5 items), treatment-related symptoms (TR; 3 items), and disease-related worries (DRW; 3 items) and one function scale (social function GINET21/SF21, 3 items) [29, 30]. With the exception of the last two questions of the QLQ-C30 (7-point Likert scale) all questions are answered on a 4-point Likert scale (ranging from “not at all” to “very much”).

According to the guidelines provided by the EORTC, all scores of the QLQ-C30 and -GINET21 were transformed linearly so that all scales range from 0 to 100. In the function scales, higher scores represents a better level of functioning. While in the symptom scales/items, a higher score marks a worsening of the symptoms [29].

### Statistics

For the purpose of this analysis, follow-up data derived by 141 follow-up questionnaires were grouped into five groups: Follow-up visit within 12 months after surgery (t1, *n* = 52 patients), within 13–24 months (t2, *n* = 33 patients), within 25–36 months (t3, *n* = 21 patients), within 37 to 60 months (t4, *n* = 11 patients) and more than 60 months after surgery (t5, *n* = 24 patients). Standard descriptive statistic measures were used to describe the study population and subgroups in regard to clinical and sociodemographic data and the QoL measures. Survival analysis was conducted using the Kaplan–Meier method, differences between survival curves were tested with the log-rank test. A *p* value of < 0.05 was considered as statistically significant.

### Ethics

All patients provided written informed consent prior to study participation. Ethical approval was achieved for the local NET-registry including the questionnaire-based interrogation by the Ethic committee of the university.

## Results

**Fig. 1:**
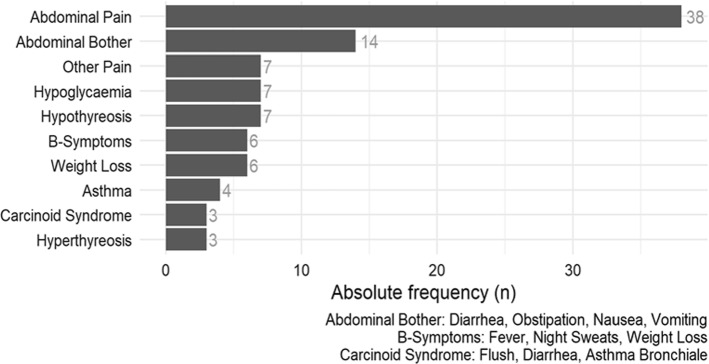
Number of patients with preoperative symptoms in NEN-patients 78 (86.7%) patients experienced at least one symptom related to NEN before surgery

**Fig. 2:**
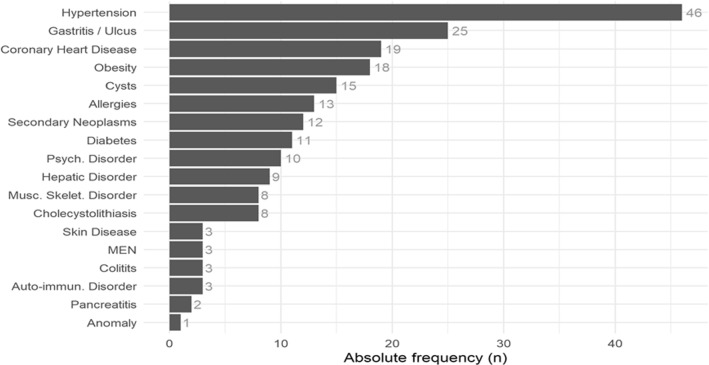
Comorbidities in NEN-patients. In 86/90 patients, at least one comorbidity was present

The study cohort (*n* = 90) consisted of 43 females and 47 males, which were selected consecutively at time of the first postoperative visit in the outpatient clinic of the department for surgery, which was usually scheduled within the first 3 month after surgery (Table 1). For preoperative symptoms and comorbidities see figure (Figs. 1 and 2).

**Table 1:** Clinical and sociodemographic data and vital status according to sex

Variable	Males *n* (%)	Females *n* (%)	Total *n* (%)
Number of included patients	47 (52.2)	43 (47.8)	90 (100)
Vital status (as of June 2015)			
Alive	40 (85.1)	39 (90.7)	79 (87.8)
Deceased	7 (14.9)	4 (9.3)	11 (12.2)
Age at diagnosis			
Mean ± SD (years)	57 ± 15	50 ± 14	54 ± 15
Tumour localisation			
Small intestine	13 (27.7)	14 (32.6)	27 (30.0)
Pancreas	8 (17.0)	10 (23.3)	18 (20.0)
CUP	6 (12.8)	6 (14.0)	12 (13.3)
Appendix	5 (10.6)	5 (11.6)	10 (11.1)
Stomach	5 (10.6)	3 (7.0)	8 (8.9)
Lung	1 (2.1)	3 (7.0)	4 (4.4)
MEN-1 (Pancreas)	1 (2.1)	2 (4.7)	3 (3.3)
Colon	3 (6.4)	0 (0.0)	3 (3.3)
Rectum	3 (6.4)	0 (0.0)	3 (3.3)
Oesophagus	1 (2.1)	0 (0.0)	1 (1.1)
Pheochromocytom	1 (2.1)	0 (0.0)	1 (1.1)
Grading	36	30	66
G1	27 (41)	26 (39)	53 (80)
G2	2 (3)	3 (7.0)	5 (8)
G3	7 (11)	1 (1.5)	8 (12)
Marital status at diagnosis			
Married	35 (74.5)	27 (62.8)	62 (68.9)
Single	7 (14.9)	8 (18.6)	15 (16.7)
Divorced	2 (4.3)	4 (9.3)	6 (6.7)
Widowed	3 (6.4)	3 (7.0)	6 (6.7)
Unknown	0 (0.0)	1 (2.3)	1 (1.1)
Occupational status at diagnosis			
Unemployed	2 (4.3)	2 (4.7)	4 (4.4)
Fulltime/part-time worker	24 (51.1)	29 (67.4)	53 (58.9)
Housekeeper	0 (0.0)	3 (7.0)	3 (3.3)
Retired/pensioner	18 (38.3)	9 (20.9)	27 (30.0)
Vocational training/study	2 (4.3)	0 (0.0)	2 (2.2)
Unknown	1 (2.1)	0 (0.0)	1 (1.1)

### Tumour stage

**Fig. 3:**
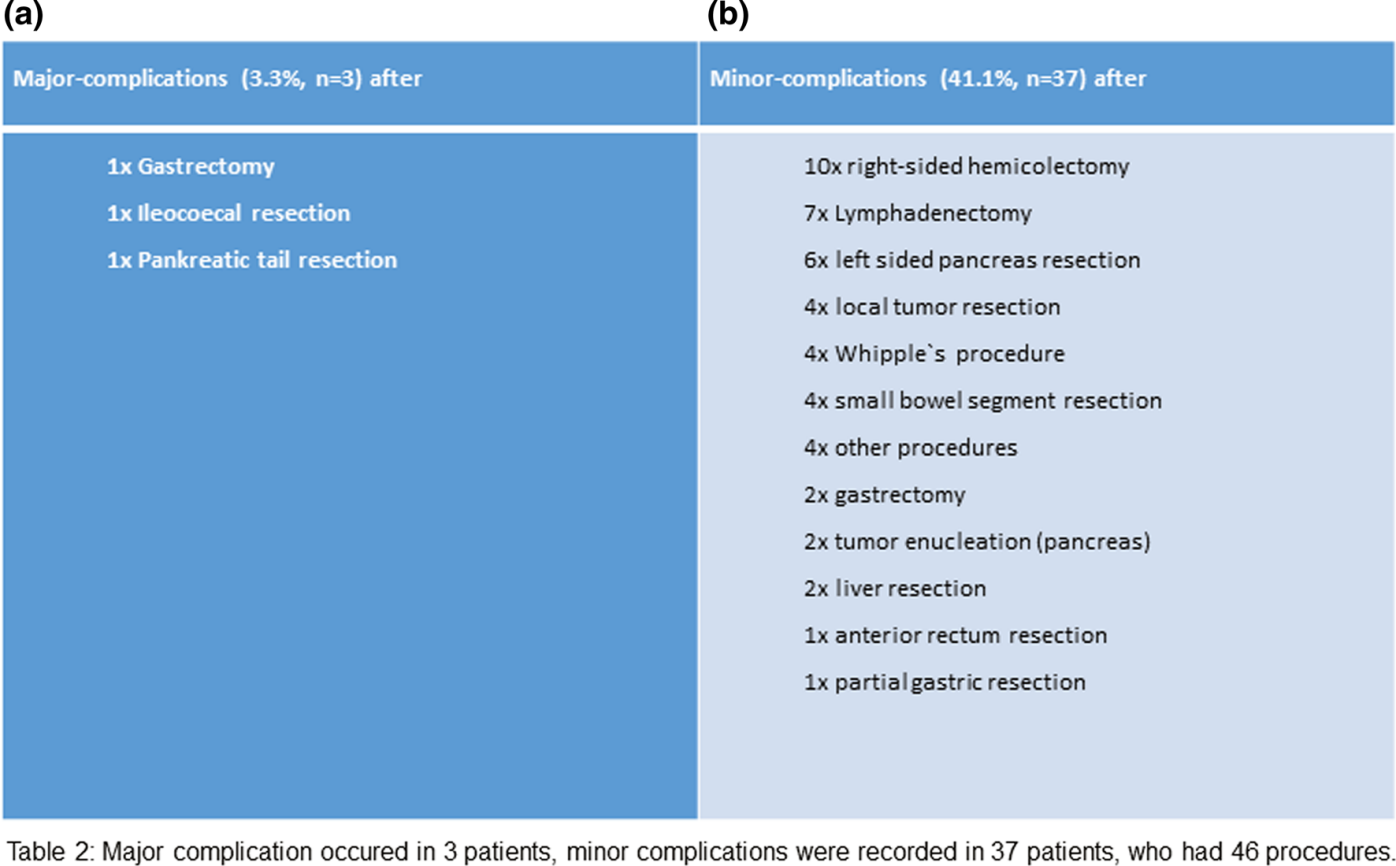
**a** Site of metastases, lymph node metastases were locoregional **b** A histological tumour free resection margin (R0) was seen in 81 patients, one patient with CUP had a macroscopic residual tumour (R2), in another patient with CUP complete tumour resection was performed, but the primary was not detected, so this patient was classified RX, in 7 patients no data concerning resection margin were available

In 45 cases no locoregional lymph node or distant metastases were seen, so this patients were grouped stage I and II, 25 patients had positive locoregional lymph node metastases without distant metastases (stage III) and 20 patients had distant metastases (stage IV). For localization of the metastases, see Fig. 3a.

### Intention of surgery and rate of complete tumour resection

**Fig. 4:**
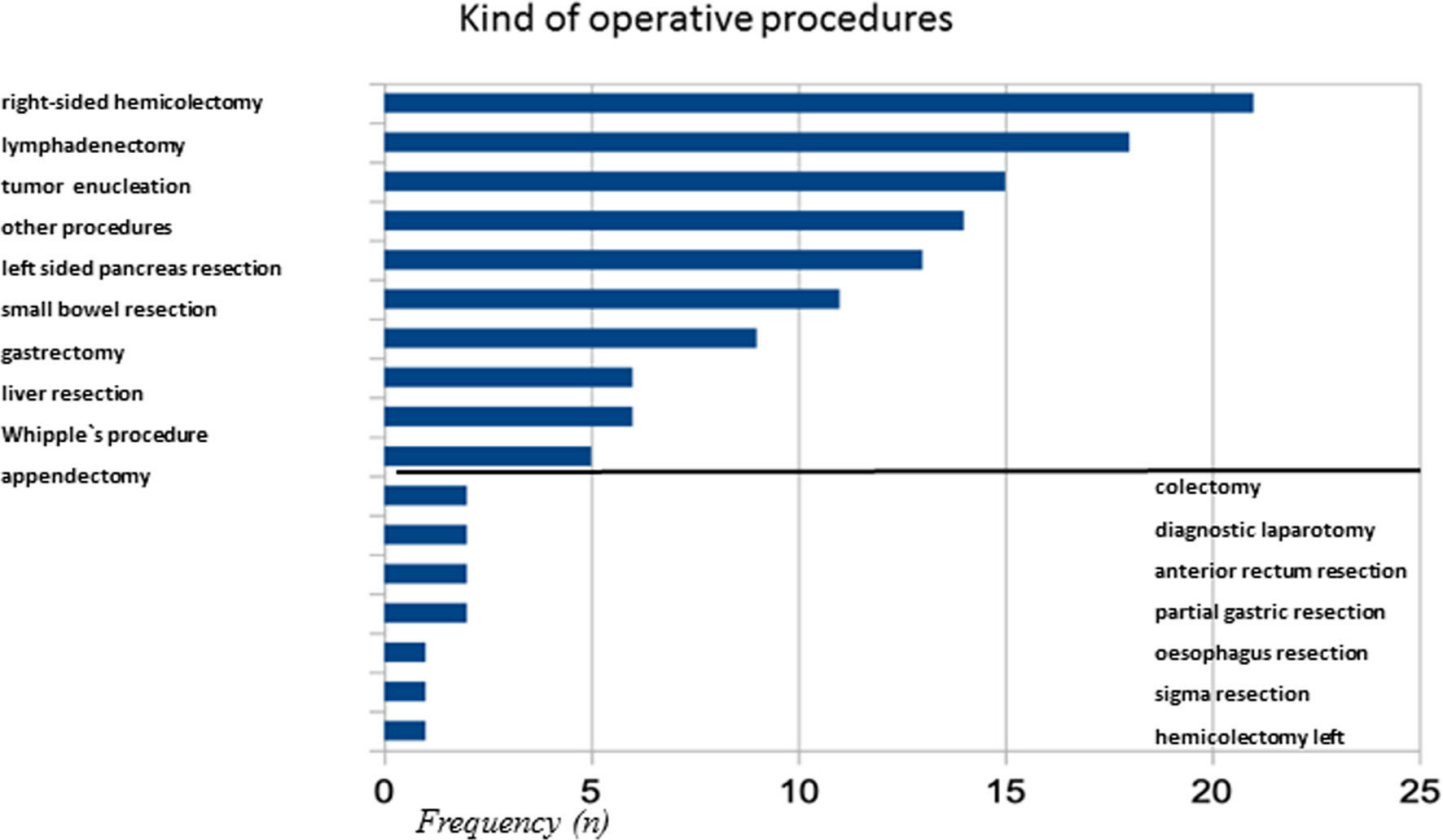
Operative procedures

All included patients received an operative procedure, which was in curative intention in 74 (82%) and in palliative intention in 16 (18%) patients. In one case of the palliative group, a diagnostic laparoscopy without resection was performed and in seven cases a complete tumour resection with histological tumour free margin was achieved in this group with initially palliative intention (Fig. 3b). The operative procedures are seen in Fig. 4.

### Tumour grading and survival

**Fig. 5:**
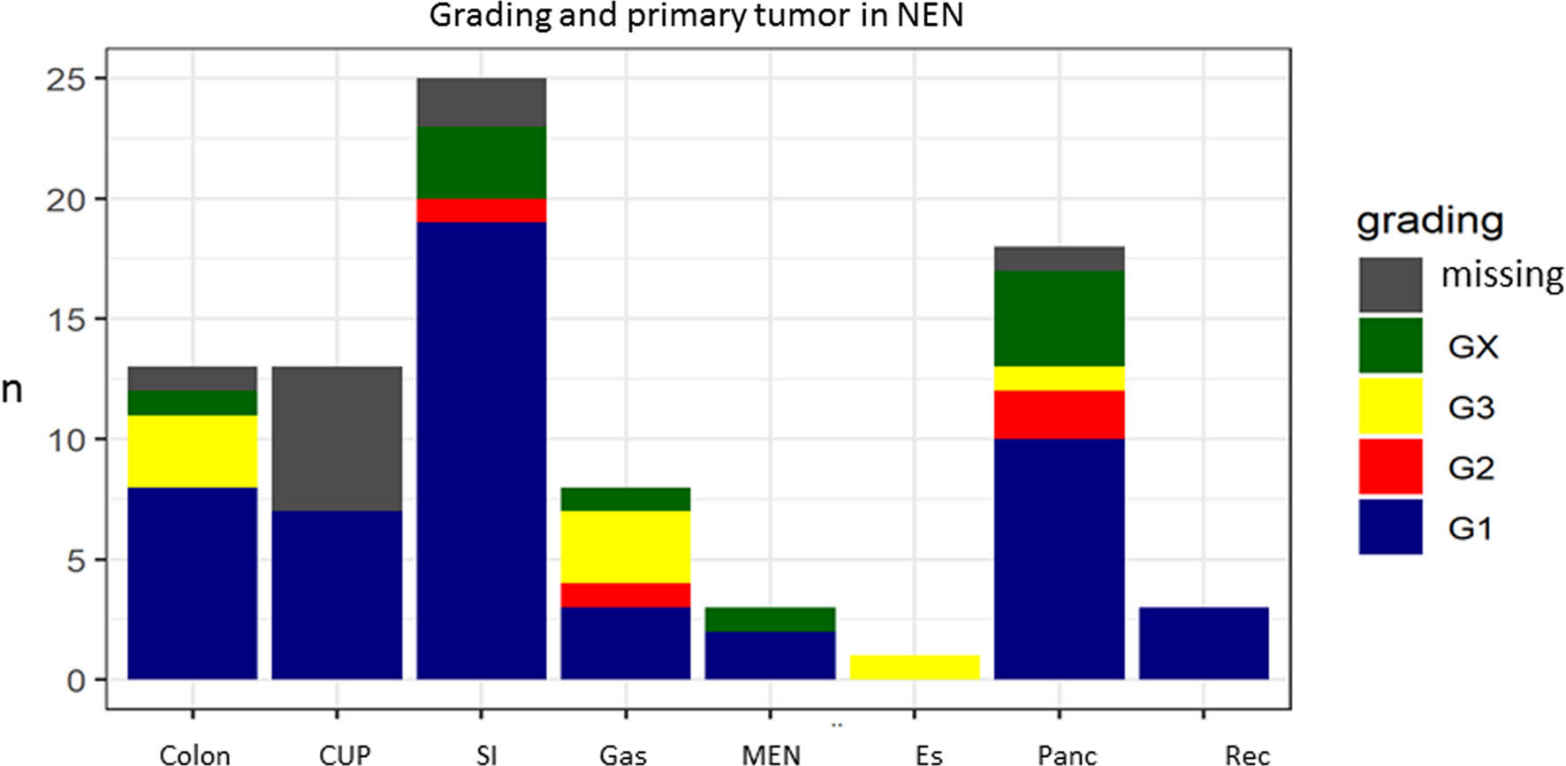
Grading according to the primary tumour. SI = small intestine, Gas = gastric, ES = oesophageal, Panc = pancreas, Rec = rectal

**Fig. 6:**
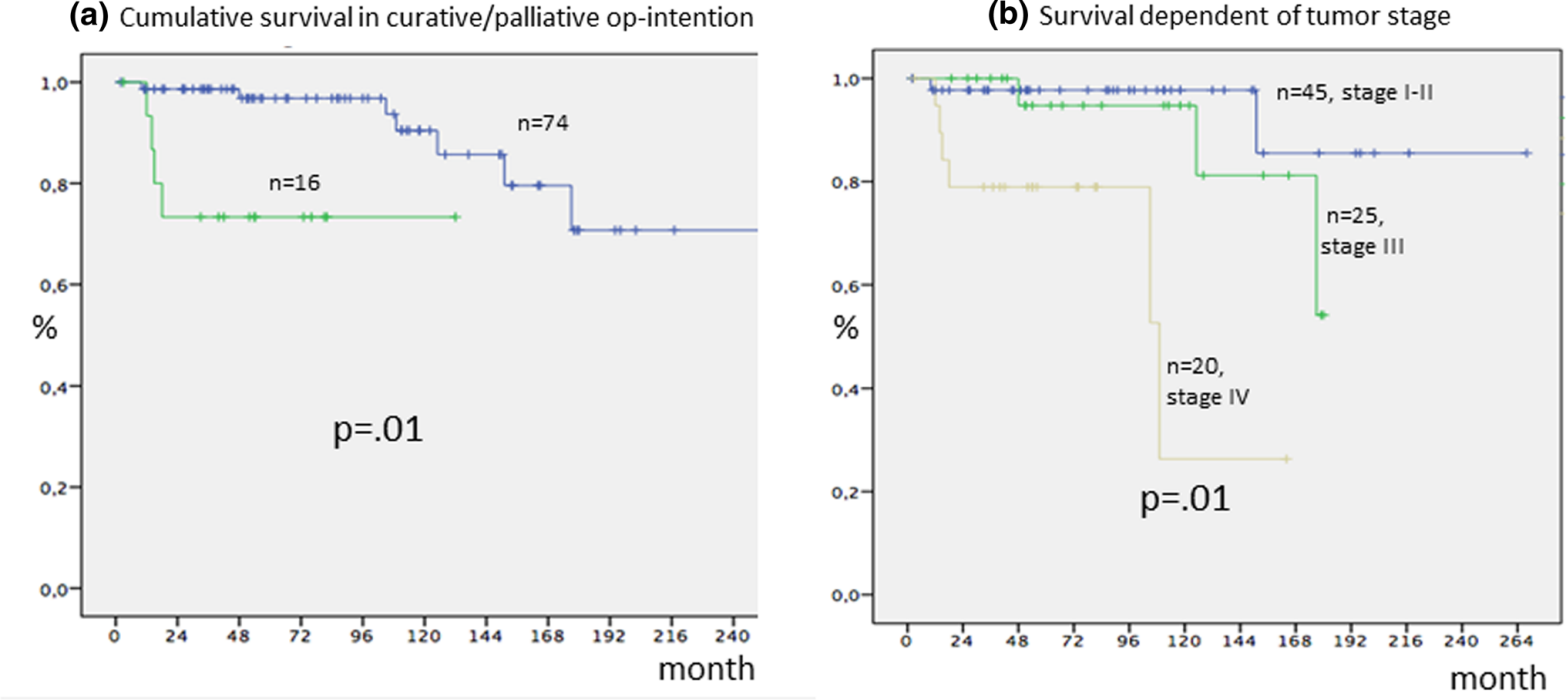
Survival dependent on **a** intention of operation (palliative vs. curative) and **b** on tumour stage: no mets. (stage I–II, *n* = 45), lymph node mets. (stage III, *n* = 34), and distant metastases in liver (18x) and or peritoneum (4x), bone (2x) and ovar (1x) (stage IV, *n* = 25)

For grading see Fig. 5. The 5- and 10-year survival rate (ysr) were 93 and 87% for all patients. In nodal negative NEN (tumour stage I/II, *n* = 45), 5- and 10-ysr were 98%. In nodal positive NEN (stage III, *n* = 25) 5- and 10-ysr were both 95%. In distant metastases 5- and 10-ysr were 79 and 26.5% (*n* = 20, *p* = 0.01, Fig. 6).

### Complications after surgery

Postoperative complications were seen in 37 of the patients (41%), in 3 cases a major complication with operative revision and in 34 patients minor-complications were seen (Table 2).

**Table 2:** Major complication occurred in 3 patients, minor complication were recorded in 37 patients, who had 46 procedures

Major-complications (3.3%, *n* = 3) after	Minor-complications (41.1%,* n* = 37) after
1 × Gastrectomy	10 × right-sided hemicolectomy
1 × Ileocecal resection	7 × Lymphadenectomy
1 × Pancreatic tail resection	6 × left sided pancreas resection
	4 × local tumour resection
	4 × Whipple`s procedure
	4 × small bowel segment resection
	4 × other procedures
	2 × gastrectomy
	2 × tumour enucleation (pancreas)
	2 × liver resection
	1 × anterior rectum resection
	1 × partial gastric resection

### Perisurgical and additional therapy

**Fig. 7:**
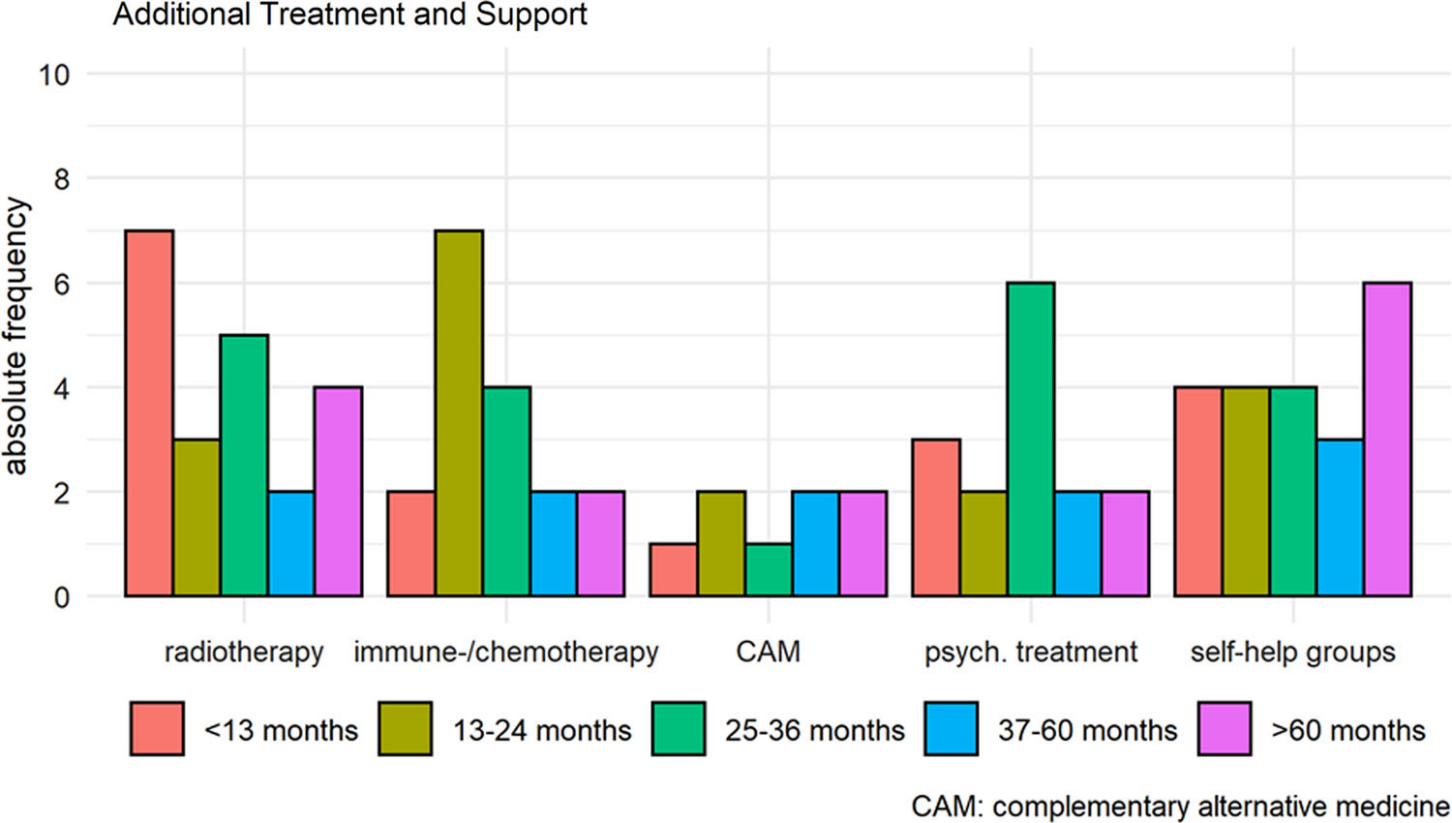
Pre- and Postsurgical therapy, alternative treatment options and support 16 patients with bio-, chemotherapy, ablation of liver metastases

One patient received a platinum-based chemotherapy before surgery for a high grade gastric cancer, initially misdiagnosed as adenocarcinoma. Seventy-four patients had no other therapy after surgery, 16 patients had a further therapy. Information on additional treatment and support were provided by 51 (t1: < 13 months), 33 (t2: 13–24 months) and 21 (t3: 25–36 months after diagnosis) patients, respectively (Fig. 7).

### Critical life events

**Fig. 8:**
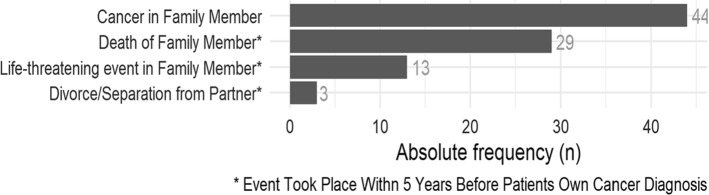
Critical life events within 5 years before diagnosis of NEN 61 patients/90 have had at least one critical life event within the last 5 years

Critical life events within 5 years before the diagnosis of NEN were reported by a total of 61 patients (Fig. 8).

### Hads-D

**Fig. 9:**
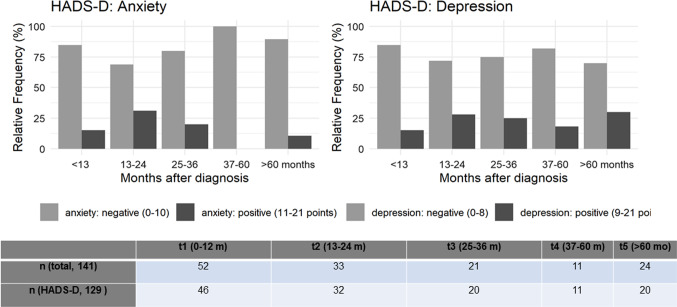
Results of hospital anxiety and depression scale (HADS-D) over time (t1-t5)

HADS-D was completed 129 times. Pathological scores for anxiety (> 10 points) increased from 20% at t1 to 30% at t2 and decreased to 10% at t5 (5 years after surgery). Pathological scores for depression (> 8 points) increased from 25% at t1 to 28% at t2 and remained stable with 30% at t5, i.e. 5 years after treatment (Fig. 9).

### EORTC QLQ-C30 and -GI-NET.21

For the EORTC QLQ-C30 and GI-NET.21 symptom scales a value of 0 means best (i.e. the absence of symptoms), 100 means worst symptoms. In the function scale 0 means worst, 100 means best function, a relevant difference is seen with 10 points difference.

EORTC QLQ-C30 is available 44 × at t1, 23 × at t2 and 14 × at t3.

**Fig. 10:**
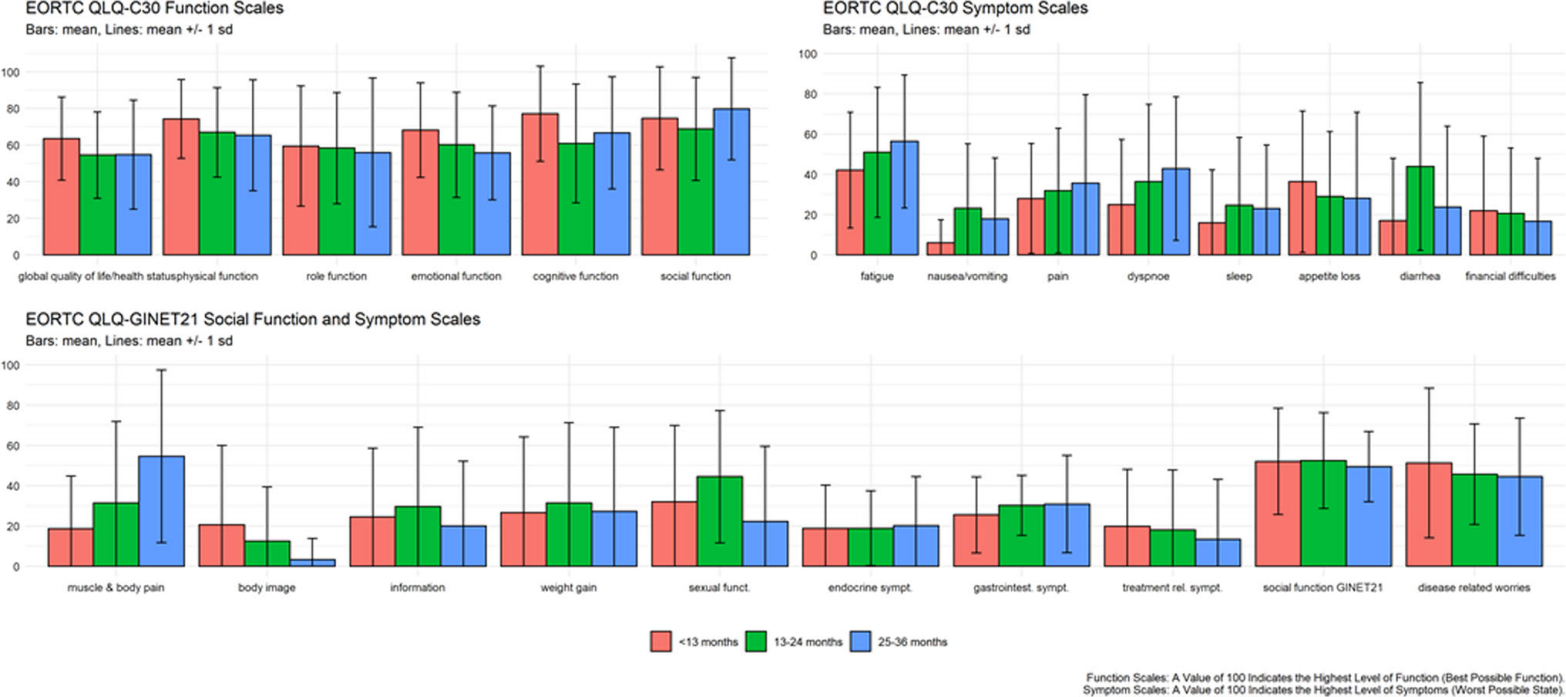
Results of EORTC QLQ-C30 and –GI-NET.21 over time (t1-t3)

The six function domains are global quality of life/health status, physical role, emotional, cognitive and social function. Scores on all function domains decreased between t1 and t3, with the exception of cognitive and social function (C30) for which scores at t3 are higher than at t2. For the latter the highest score of nearly 80 points, and thus highest social function, is reached 25–36 months after surgery. Emotional and cognitive function showed a decrease of more than 10 points, which means a clinical worsening (Fig. 10).

The nine symptom items/scales in the QLQ-C30 questionnaire were fatigue, pain, dyspnoea, diarrhoea, nausea and vomiting, constipation, loss of appetite, sleep and financial difficulties. Fatigue, pain, dyspnoea, appetite loss and diarrhoea are among the symptoms that were considered most troublesome as indicated by high scores. An initial increase (between t1 and t2), but a further decrease in symptoms (between t2 and t3) is observed for nausea and vomiting, sleep, constipation and diarrhoea. Fatigue seem to increase in a clinical relevant manner from t1 to t3 and remains high with > 50 points (Fig. 10).

According GI-NET.21 social function showed the same level at all time points with scores around 50. A high level of bother as indicated by scores > 40 points was observed for muscle and/or body pain at t3 and disease-related worries at all time points. Clinical relevant changes were seen for muscle and/or body pain (increase in symptoms) and body image (decrease in symptoms, Fig. 10).

## Discussion

Little is known about how patients with neuroendocrine neoplasia deal with the demanding situation caused by the disease with its risk for late tumour recurrence followed by multimodal treatment after surgery. These are the first long-term data for QoL over 3 years after surgery in a mostly curatively treated cohort of NEN-patients as in 81/90 patients a complete tumour resection (R0) was achieved. In general, we see a stabilization of all functions and a decrease in symptoms over time. For endocrine, gastrointestinal and treatment-related symptoms we find ranges between 10 and 30 points 3 years after surgery, which means nearly normal situation. Fatigue shows an increasing with > 50 points and muscle/body pain with > 40 points after 3 years, so patients should be monitored for this even after curative treatment. Possible bias are the high rate of G1/G2 tumours (88%) and the low rate of G3-NEC (12%) resulting in high 10-years-survival rates (98, 95 and 79% for stage I/stage II, stage III, stage IV). The impact of distant metastases at time of diagnosis is seen after 10 years with a survival rate of 26%, only (*p* < 0.001, Fig. 6). Another limitation of our study is, that we did not differ the outcome of the questionnaires between patients with and without tumour persistence or tumour recurrence. Another limitation of our study is the lacking comparison group, which could be other cancer survivors like colorectal carcinoma. More homogeneous groups and a possible influence of gender should be analysed in the future studies.

### Quality of life

Watson et al. published a systematic literature review concerning QoL in NEN-patients: 43 publications were eligible: 8 in GI-NET only and 6 studies in pancreatic NEN. Most commonly, EORTC-QoL-C30 was used, 6 studies used the additional battery of GI-NET21. Not surprisingly, there was no surgical cohort and the result was, there was no difference between placebo and treatment arm (biotherapy, chemotherapy, molecular-targeted therapy and PRRT) within 12 month except in the NETTER-1-study, in which the active treatment improved QoL [31, 32]. Milanetto et al. published a study with 100 patients with Si-NET after surgery and found male gender, younger age, treatment with Somatostatin analogues, non-symptomatic tumour and small intestinal surgery associated with better quality of life [33]. Karpinnen et al. studied QoL in 134 SI-NEN-patients with SF-36 and 15D and compared this to the general Swedish population (*n* = 1153). The study population differed from our concerning primary (SI only), 91% had a metastatic situation, around 79% received biotherapy (SSTRA). They found an overall impairment, especially for diarrhoea, sleep, depression, vitality and sexual activity. Interestingly, they found a correlation with number of medication and a high prevalence of depression in carcinoid syndrome with 35–50% [34].

According to Larsson et al. patients with endocrine GI tumours enjoy a relatively good quality of life, which we could not find in our cohort [35]. Compared to the data of the EORTC QLQ-C30 of the German population the NEN-patients have worse function and aggravation of symptoms in nearly every domain [36]. The impairment of QoL was lasting during the follow-up period and showed no tendency towards normalization. Scores on all functional domains decreased over 3 years after surgery, representing a worsening of function. Fatigue is a general problem in patients with cancer diagnoses [35]. According to Bower et al. approximately 20% of the cancer survivors report persistent fatigue after curative treatment, which we find in our cohort, too [37]. It is noteworthy that this remains a problem in a patient cohort of which 90% had a curative tumour resection.

### Anxiety and depression

The prevalence of psychiatric disorders at time of diagnoses in NEN in our cohort is high with 12% (10/90) but comparable to other cancer cohorts such as breast cancer. History of major depression in a small series of breast cancer patients was reported in 10% [38]. Zhu et al. found in only 4.49% of cancer patients previous mental disorder in a Swedish population of 218.000 cancer patients. The cancer stage was comparable to our group with advanced stages only in 8% [23]. According to Mitchell et al. the rate of depression is 2–4 times higher in cancer patients compared to normal population [39]. They found around 30–50% of patients in cancer suffering from psychiatric disorders. The rate of anxiety and depression according to HADS-D was high in the postsurgical period. Anxiety shows the tendency towards normalization, but scores for depression remain high with 30% during follow-up. Lewis et al. found a pathological anxiety score (> 8/21) in 26% and a pathological depression score (> 8/21) in 10% in a group of 50 patients with advanced NEN [40]. The history of major depressive disorder is a general risk factor for poor psychological adjustment to stress, including a cancer diagnosis and may also influence cancer-related fatigue and outcome [37, 41] Sleep disturbance is common in cancer patients and survivors and is strongly correlated with fatigue, too [42].

The rate of critical lifetime events seems to be very high within 5 years before diagnosis of NEN. So far, there is no known causality between this and the onset of diagnoses of NEN. But further studies with comparative groups are needed to shed light on this finding.

## Conclusion

Quality of life in patients with NEN in a group with high rate of curation remains stable after surgery and shows no tendency to worsening within 3 years of follow-up. Fatigue and muscle/body pain remain symptoms with clinical relevance, though. Anxiety decreases over time, but the rate of depression remains high with 30% in our cohort. Studies with focus on the long-term effects of surgery in this complex patient group are necessary. As depression and psychiatric disorders can reduce cancer survival due to different reason, a clinical focus should be on diagnosing the patients at risk and refer them to further treatment. HADS-D is an appropriate instrument in NEN-patients for screening for anxiety and depression and should be implemented into the follow-up even after curative resection. The authors see an urgent need for monitoring quality of life and mental health as important clinical oncological issue also in a surgical cohort.
